# Application of Polysaccharide Biopolymer in Petroleum Recovery

**DOI:** 10.3390/polym12091860

**Published:** 2020-08-19

**Authors:** Shunxiang Xia, Laibao Zhang, Artur Davletshin, Zhuoran Li, Jiahui You, Siyuan Tan

**Affiliations:** 1 Department of Petroleum and Geosystems Engineering, University of Texas at Austin, Austin, TX 78712, USA; davletshinar@utexas.edu; 2 Independent Researcher, Baton Rouge, LA 70820, USA; laibaozhang21@gmail.com; 3 Department of Petroleum Engineering, University of Houston, Houston, TX 77023, USA; zli38@uh.edu (Z.L.); jyou9@uh.edu (J.Y.); 4 Department of Civil Engineering, New Mexico State University, Las Cruces, NM 88003, USA; tan@nmsu.edu

**Keywords:** biopolymer, petroleum, oilfield chemicals, viscosity, drilling fluid, fracking fluid, enhance oil recovery, microbial plugging, wastewater

## Abstract

Polysaccharide biopolymers are biomacromolecules derived from renewable resources with versatile functions including thickening, crosslinking, adsorption, etc. Possessing high efficiency and low cost, they have brought wide applications in all phases of petroleum recovery, from well drilling to wastewater treatment. The biopolymers are generally utilized as additives of fluids or plugging agents, to correct the fluid properties that affect the performance and cost of petroleum recovery. This review focuses on both the characteristics of biopolymers and their utilization in the petroleum recovery process. Research on the synthesis and characterization of polymers, as well as controlling their structures through modification, aims to develop novel recipes of biopolymer treatment with new application realms. The influences of biopolymer in many petroleum recovery cases were also evaluated to permit establishing the correlations between their physicochemical properties and performances. As their performance is heavily affected by the local environment, screening and testing polymers under controlled conditions is the necessary step to guarantee the efficiency and safety of biopolymer treatments.

## 1. Introduction

Polysaccharide biopolymers are regarded as biomacromolecules derived from renewable resources, in a raw or chemical modified form [1]. They are advantageous for a wide range of applications in the industry of food, pharmaceutics, cosmetics, construction, chemicals, textiles, etc., due to their versatile physical behaviors, multiple functions, relatively low price, sustainability and environmental safety [2,3]. The global capital market of biopolymers is expected to reach USD 10 billion by 2021, with a 17% annual growth rate [4]. Since the inception of the petroleum industry, polysaccharide polymers have been used in almost every section of the petroleum industry to reduce the recovery cost and enhance operation efficiency. Besides generally requiring polymer as reliable and effective, the petroleum industry emphasizes more on the economy of polymers, especially when oil and natural gas are at a low commodity price. Therefore, instead of ultra-pure polymers, the polymers in their naturally occurring composite forms, with little or no modification, are preferred by the oil and gas industry operations [5]. 

**Figure 1:**
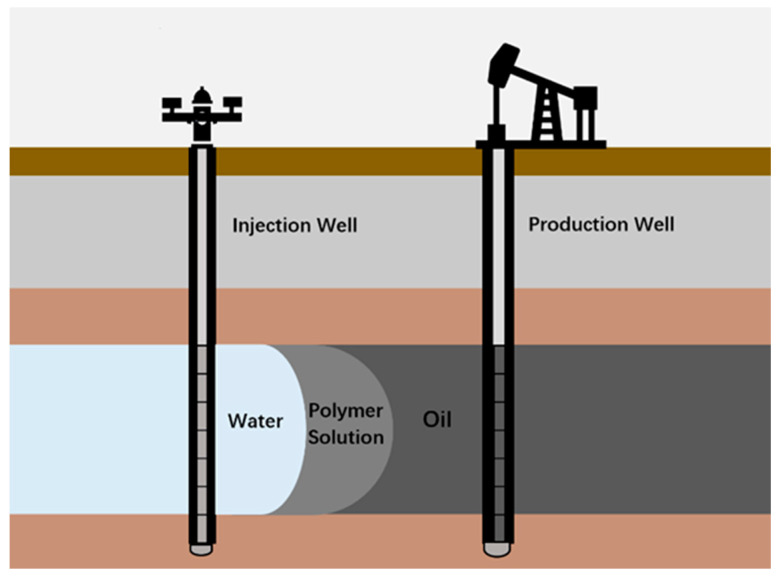
The scheme of traditional petroleum recovery process with polymer flooding.

For operators, understanding the workflow of petroleum recovery, as well as the property requirements of biopolymers in the process, is the prerequisite for designing efficient polymer treatments. As shown in Figure 1, petroleum is one kind of hydrocarbon fluid that exists in the connected pore space of a reservoir, and it is typically extracted through oil wells. To initialize the oil production, oil wells are drilled from the surface to reservoirs with various deepness between 2500 and 20,000 feet. Once the well is complete, an oil flow occurs due to the pressure difference between the reservoir and wellbore, which can be expressed as:

(1) $$Q=-\frac{kA}{\mu L}\nabla p$$

where *Q* is the oil flow rate, *k* is the permeability, μ is the viscosity of oil, *A* is the cross-sectional area of wellbore, *L* is the reservoir distance, and ∇p is the total pressure drop. 

Especially, when the permeability (k) of the reservoir is too low to support a significant rate of oil flow, hydraulic fracturing will be a common approach to enhancing the flow rate by increasing the cross area of the wellbore. During the early stage of production, the recovery is called primary production and it is driven by the reservoir’s natural energy such as fluid and rock expansion, solution-gas drive, and gravity drainage. Generally, only ~10% of the original oil in place (OOIP) can be recovered through the primary production, along with a gradient descent of reservoir pressure. Once the decreasing reservoir pressure cannot afford oil flow, water is injected from injection wells into the formation, increasing the reservoir pressure to its initial level, and the oil recovery enters the secondary recovery domain. During this period, the water displaces oil from the pore spaces, and is produced in the form of oily water associated with the oil production. The water flooding generally brings a 15–25% incremental OOIP recovery, and the increment is heavily affected by a series of factors including oil viscosity, formation permeability, connate water compatibility, rock wettability, etc. To further seize the residue oil, enhanced oil recovery (EOR) may be conducted depending on the local geological conditions. Based on the various mechanisms of changing fluid properties, prevailing EOR approaches include thermal, chemical (surfactant and polymer) and miscible methods. Especially, the polymer method is widely applied to improve the mobility ratio and divert injected water from zones that have been swept. The EOR approaches can realize an overall 30–60% recovery of OOIP. However, it also significantly increases the cost of development. To ensure a successful petroleum recovery project, the economics of the development equation, as well as environmental concerns about wastewater recycling, must be considered. In that case, utilizing polymers, especially biopolymers, as fluid additives, is an important aspect regarding the efficiency and safety of petroleum recovery. For example, Xanthan gum, Scleroglucan and cellulose and their derivatives are used as thickness agents in the drilling and production process; Guar gum and Guar gum derivatives are applied to form crosslinked gels for proppant transportation in hydraulic facture; Chitosan is utilized as oil adsorbent in wastewater treatment, etc. Besides biopolymers, synthetic polymers such as polyacrylamide (PAM) and hydrolyzed polyacrylamide (HPAM) are also used as thickening agents during flooding and plugging process. These acrylamide-based polymers possess larger viscosity due to the repulsion forces among their negatively charged linear chains. As a result, the viscosity is negatively affected by the cations in the environment. While synthetic polymers generally exhibit simple and homogenous structures, their high cost, complicated synthetic process, and potential toxicity to the environment limit their application realms in the petroleum recovery process.

Compared with synthetic polymers, biopolymer possess several advantages. Biopolymer can provide a super thickening effect at a lowest cost. For example, a 1% Guar gum solution can hold the viscosity of up to 10,000 mPa·s and adequate stability against shear stress and temperature. The price of Guar gum can be as low as USD 2/kg due to its abundant resource and simple extraction process. The excellent thickening effect, as well as low price, puts Guar gum in a dominant position on the market of hydraulic fluid additives. Moreover, as macromolecules, the flexible molecular structure and active groups of biopolymers provide enough room for property modification, allowing the versatile functions of biopolymer in the petroleum recovery process. Finally, as natural products, biopolymers are non-toxic and environmentally friendly, and their residue has less impact on local health and safety. In contrast, the increasing concerns about the long-term safety of using (H)PAM in fluids has risen around the world. Although (H)PAM is non-toxic to animals and plants in the form of polymer, many studies have proven that the degradation of the polymer, caused by mechanical stress and harsh chemical/thermal conditions, can generate acrylamide monomers, which are neurotoxin and potential carcinogen [6]. The monomer is highly mobile in the environment regarding its high solubility in water, and has brought environmental challenges, both in water management and in contamination of local water supply. To address this issue, many institutions around the world have established strict regulations on the application of (H)PAM. For instance, the U.S. Environmental Protection Agency (EPA) and European Commission (EC) have a limit of 0.5 ppb and 0.1 ppb (*w*/*v*) acrylamide in production water, respectively [7].

Along with various useful characteristics, the features of petroleum industry also bring several challenges to the application of biopolymers. First, the performance of biopolymers is heavily affected by the geological conditions of reservoirs. Many factors including temperature, pressure, permeability and mineralogy of the reservoir, as well as the composition and rheology properties of formation fluids, need to be evaluated for a successful application of polymer. However, the acquisition of these parameters is unfeasible or too costly sometimes, and therefore the design of polymer treatment may confront several unknown parameters. Moreover, although laboratory tests can reveal some pieces of evidence about the positive effort of biopolymers, the limited field data make the actual influence of polymer on petroleum recovery imperceptible for operators. The lack of data also makes it hard to adjust the recipe of biopolymer during the treatment for better performance. Finally, even the benefits of polymer flooding exist, the improvement usually needs several months and even years to be recognized and evaluated [8], and delayed and undetermined improvement would bring reluctance and refusals of operators to the polymer treatment.

In this review, a bridge between biopolymer chemistry and petroleum recovery process was established by investigating both the characteristics of biopolymers and the requirements of physiochemical properties of fluids in the crude oil extraction process. The benefits and limitations of biopolymer as fluid additives are discussed to provide insight to the potential improvement of current petroleum recovery process. While the biopolymer treatment is usually on a case-by-case basis, this work provides general guidelines of biopolymer application in the petroleum recovery process, aiming at realizing a greener and more efficient energy production.

## 2. Polysaccharide Biopolymer

Polysaccharide biopolymer contains monomeric sugars linked together with O-glycosidic linkages to form a larger structure. The properties of monomers, linkages and potential chemical modifications collectively determine the characteristics of polymers. Before the beginning of the petroleum industry, biopolymers had already been applied widely in the food and pharmaceutical industry and emerging petroleum recovery technologies brought additional opportunities to the development of biopolymers. Regarding the cost, most biopolymers applied in petroleum engineering are produced from the direct extraction of raw plant materials or large-scale fermentation, and lots of effort has been done to increase their production yield. Moreover, as the functions of polymers lie on their molecular structure and conformation, modifying their molecular structure is a prevailing method to improving their physicochemical properties. Table 1 summarizes the typical biopolymers involved in petroleum recovery, and the detail is discussed as follows.

### 2.1. Xanthan Gum

Xanthan gum was named after the Xanthan-producing bacterium *Xanthomonas campestris*, and it was discovered at the Northern Regional Research Laboratories of the United States in 1950 [57]. As a polysaccharide polymer, Xanthan gum has attracted wide attention from the industry due to its high solubility, thermo-stability, high viscosity yield and gelation capacity, and it plays versatile roles as thickener, stabilizer, dispersant, fat replacer and coating material. The food industry consumes 60% of the global production of Xanthan gum, followed by oil industry (15%) and others include Medical, Personal Care, Cosmetics industries, etc. Due to the high demand, the market capitalization of Xanthan gum increases at a 5–10% annual rate and reached USD 722 million in 2016 [58]. Nowadays, the major producers of Xanthan gum include Merck and Pfizer in the United States, Fufeng Group and Deosen Biochemical in China, Rhône Poulenc and Sanofi-Elf in France, and Jungbunzlauer in Austria, with an average market price of USD 12/kg [9].

**Figure 2:**
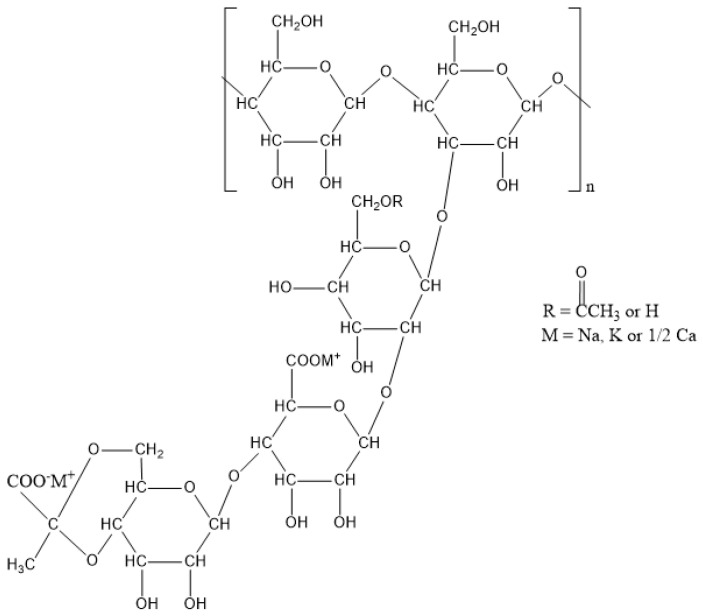
The molecular structure of Xanthan gum.

Xanthan gum is an anionic heteropolysaccharide with a linear β-(1–4)-d-glucopyranose glucan backbone, and the C–3 position of every other glucose links a trisaccharide side chain containing d-mannose, d-glucuronic acid, and d-mannose in an order (Figure 2). The pyruvic acid residue linked to the terminal d-mannose and the acetyl group linked to the d-mannose unit collectively bring the negative charges to Xanthan gum [10]. The molecular weight (*M*_W_) of Xanthan gum is around 2 × 10^6^ to 2 × 10^7^ Da, and the stiffness, as well as the intermolecular associations, of its molecules enables Xanthan gum to exhibit a shear-thinning effect [11]. The viscosity of 1 g/L Xanthan gum is between 13–35 mPa·s and the viscosity is stable at low pH values (up to pH 3), high salinities (up to 3% salt) and temperatures (up to 80 °C) [10]. However, it may still lose the viscosity because of the denaturing process occurred at too high temperature coupled with low ionic strength when its structure appears as disordered and flexible coils. The denature is reversible as the coil structure can be turned back to be an ordered single or double helix conformation by lowering the temperature or increasing the ionic strength [12]. The rheology and stability of Xanthan gum are determined by the value and distribution of the *M*_W_, as well as the components of polysaccharides, which are varied based on the producing bacterium species, fermentation condition and separation process. To increase its velocity, crosslinking may be implemented in the application of Xanthan gum, which is triggered by adding Ca^2+^ ions [13], adipic acidic dihydrazide or sodium trimetaphosphate [14].

**Figure 3:**
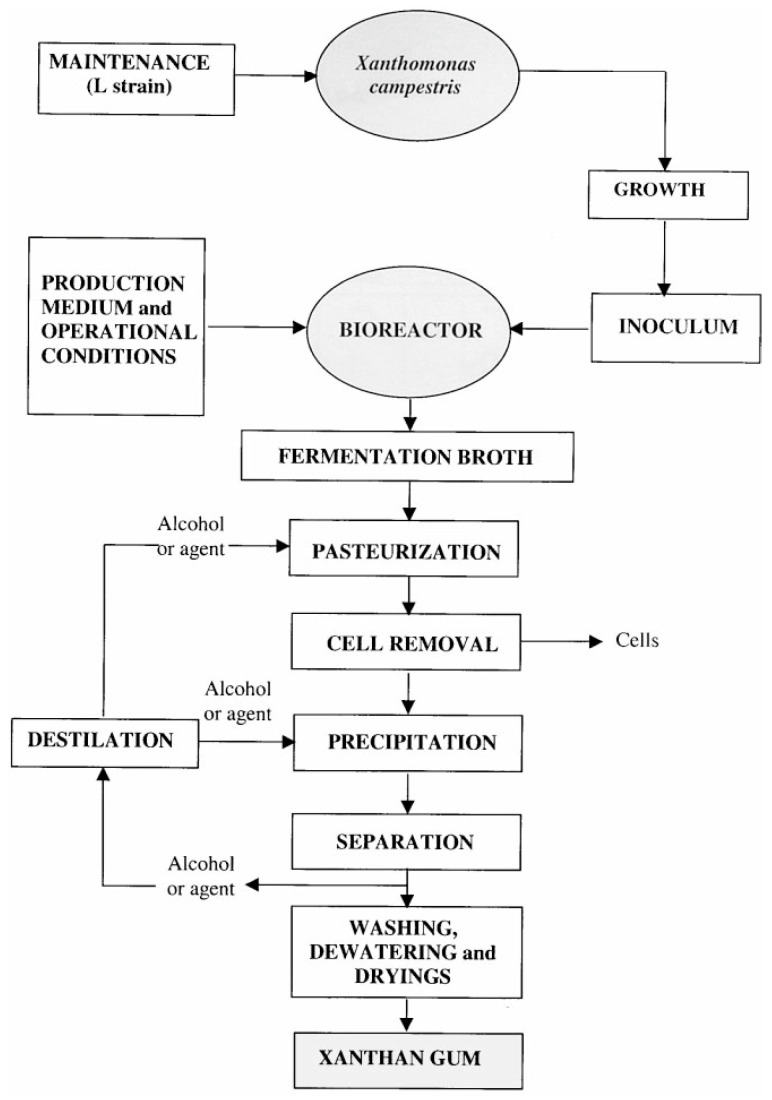
The production process of Xanthan gum, reproduced with permission from [10]; copyright Elsevier, 2000.

Xanthan gum is usually manufactured through a traditional fermentation–purification process, shown in Figure 3. Xanthomonas, a genus of *Pseudomonadaceae*, is fermented with a production medium in a bioreactor to produce Xanthan gum, which constitutes the bacterial capsule. Numerous effects have been carried out to optimize the fermentation process to maximize the yield and reduce the cost. For example, with a 200–300 rpm and 1 L/min air flow rate, the production medium was optimized with 2–4% glucose or sucrose for carbon source [59] and 15 mM of glutamate [60] for nitrogen source to maximize the yield. Shu and Yang pointed out that the temperature control improved the quality and quantity of Xanthan gum production: the highest yield was observed at the temperature between 31–33 °C, but 27–31 °C was preferred for producing Xanthan gum with a high pyruvate content [61]. In contrast, the pH control of the medium only affected the cell growth instead of Xanthan production [62]. The fermentation usually lasts around 50 h, and the concentrations of Xanthan, cells and residual nutrients in final fermentation broth are 10–30 g/L, 1–10 g/L and 3–10 g/L, respectively [15]. The high content of impurities and viscosity make the purification process extremely complicated and time-consuming. The purification step begins with a thermal treatment, with which the broth is kept at 80–130 °C and pH 6.3–6.9 for 20 min to increase the solubility of Xanthan gum. Then, the broth is diluted with water to reduce the overall viscosity, followed by the first filtration to remove the cell debris from the broth. Finally, lower alcohol (≥6 vol per broth volume) and salts are added into the broth to trigger the precipitation of Xanthan gum, which is recovered by the second filtration.

To meet the rheology and solubility needs of the petroleum industry, chemical modifications of Xanthan gum are widely applied to improve the thickening effect, solubility, and thermal stability. The general approaches include carbonate-modified, formaldehyde-modified, and propylene oxide-modified. Aiming at improving the thickening effect of Xanthan gum, Reddy conducted the carbonate-modification of Xanthan gum and the results are shown in Table 2 [16]. The carbonate-modified Xanthan gum was synthesized by mixing the Xanthan gum and various organic carbonates with the mass ratio ranged from 1:0.1 to 1:20. Then, the mixture was heated at 70 to 80 °C in a roller oven for 6 to 24 h to complete the modification. As shown in Table 2, carbonate-modifications significantly increased the *M*_W_, as well as the viscosity, of Xanthan gum. Among them, the modification with glycerine carbonate exhibited the highest improvement, as doubled viscosity was achieved. The author also investigated the effect of modification on the stability of Xanthan gum against high temperatures. When the temperature was shifted from 80 °C to 121 °C the ethylene carbonate modified Xanthan gum exhibited an increasing viscosity, different from a decline in viscosity with untreated Xanthan gum. The thickening effect of Xanthan gum can also be improved with hydroxypropyl modification, which is achieved by reacting Xanthan gum with propylene oxide under an alkaline condition. According to the research conducted by Tian et al., the hydroxypropyl modification possessed a 25% increase in thickening effect without affecting the drag-reducing effect [17]. Besides the thickening effect, a quick dissolving of Xanthan gum in a fluid is also expected to avoid the plugging issues during the injection. To address this concern, Su et al. conducted the formaldehyde-modification of Xanthan gum and decreased the dissolution time of Xanthan gum from 30 min to 8 min. In the study, Xanthan gum (3 wt%) was mixed with formaldehyde at 100:1 volume ratio under pH 1.6~2.0, and mechanically stirred at 40 °C for 6 h to generate formaldehyde-modified Xanthan gum [18]. Besides the modifications mentioned above, deacetylated [63] and hydrophobic modification [64] were also reported to improve the viscosity Xanthan gum.

### 2.2. Scleroglucan

Scleroglucan is the extracellular polysaccharide, as a form of restored energy, of the fungus of the genus *Sclerotium*. After Halleck discovered Scleroglucan from fungus *Sclerotium glucanicum* in the 1960s [65], Scleroglucan has been introduced to the market as a food viscosity agent and texture modifier under the trade name Polytran by Pillsbury Co. Its great thickening effect and its stability with wide temperature and pH have attracted the attention of petroleum engineers since the 1970s. Scleroglucan has been widely used in the section of enhanced oil recovery of the petroleum industry, where Scleroglucan is added into the injection water to increase the viscosity and improve the oil recovery. The market price of Scleroglucan is around USD 50/kg and it is produced by many great global producers such as Cargill, ELICITYL, General Mills, Carbosynth, Shandong Qilu Biotechnology Group, etc.

**Figure 4:**
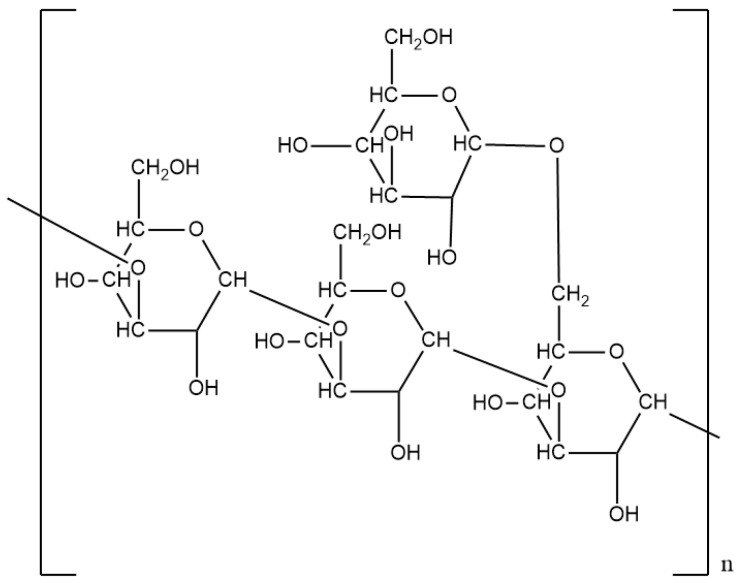
The molecular structure of Scleroglucan.

Scleroglucan has a main linear chain of β-d-(1–3)-glucopyranosyl units and a (1–6)-linked β-d-glucopyranosyl side unit is linked at every third main chain unit (Figure 4). The *M*_W_ of Scleroglucan is ranged from 1.3 × 10^5^ to 6.0 × 10^6^ Da, varied from different producing strains and fermentation conditions [19]. Scleroglucan disperses easily in the water due to the presence of glucopyranosyl group [66], and 35 mg/L of Scleroglucan can give 10 mPa·s of viscosity. Under low concentration, Scleroglucan solution can be treated as a Newtonian fluid, while the solution exhibits a pseudoplasticity behavior when Scleroglucan is more than 0.2 wt%. The excellent thermo-stability of Scleroglucan comes from its rigid triple-strand helix conformation, yet the large fluctuates of temperature or pH can still denature the polymer through interrupting the hydrogen bonds between the strands of polysaccharides. Self-supporting sliceable gels were observed in a 1.2–1.5 wt% Scleroglucan solution at 25 °C and adding 0.15 wt% bentonite can generate viscous gel with the viscosity more than 4000 mPa·s [20]. Generally, Scleroglucan possesses better stability than Xanthan under extreme environments, which is a common concern in petroleum production. Kalpakci et al. studied the thermal stability of Scleroglucan at realistic reservoir conditions and found only 10–20 percent of the original viscosity was lost at 105 °C after 460 days. Moreover, the Scleroglucan solution can retain all of its viscosity at 100 °C during the two years research period [21]. Scleroglucan is recommended to be applied at temperature of up to 135 °C while the loss of viscosity occurs in a short time beyond this threshold [22]. Besides the ideal thermostability, Scleroglucan also manifested high tolerance of alkali condition; as indicated by Kashiwagi et al., Scleroglucan can keep stable in the 0.02 M NaOH (pH 12.3) at 25 °C [23]. The outstanding rheology and stable properties make Scleroglucan the second-largest utilized biopolymer in the petroleum industry, following Xanthan gum.

Aerobic fermentation of *Sclerotium rolfsii* is the commercial approach to producing Scleroglucan. The production is favorable with a high carbon to nitrogen ratio, and Valdez et al. recommended 30 g/L sucrose and 3 g/L NaNO_3_ for the carbon and nitrogen source, respectively [67]. The addition of phosphorus, sugar nucleotides and amino acids enhanced the metabolism pathway of Scleroglucan synthesis and had a positive impact on the production yield. The optimum fermentation temperature is 28 °C and the optimum pHs for cell growth and polymer production are 3.5 and 4.5, respectively. The limited oxygen supply was proven to improve the production of polymer while inhibiting the cell growth [68]. After 72 h of fermentation, the concentration of Scleroglucan in the broth can reach 10–20 g/L. To purify Scleroglucan, alkali and water are added to the broth for neutralization and dilution, then the broth is heated at 80 °C for 30 min, homogenized and centrifuged to remove cell debris and residue substrate. Finally, Scleroglucan is precipitated and recovered from the broth by adding alcohol, CaCl_2_ and adjusting the pH [69].

The study of hydrophobic modification of Scleroglucan has been conducted in pilot-scales, aiming to reduce the interfacial tension between oil and water, which can enhance the oil recovery [24]. To obtain amphiphilic Scleroglucans, various densities of hydrophobic stearate groups were grafted onto the triple-helix conformation of Scleroglucans. After that, ionic-sulfonic groups were also attached to the polysaccharide to generate polyelectrolyte. With 0.3 w/v% of StCl(0.3)– Scleroglucans–SO_3_–, the interfacial tension was decreased from 0.105 to 0.035 N/m. The hydrophobicity of the polymer was also helpful in reducing the adsorption of the polymer during oil displacement. While chemical modifications bring some new characteristics to Scleroglucan, the treatments are considered as economically infeasible for most petroleum projects due to the high cost.

### 2.3. Guar Gum

Guar gum is a natural non-ionic polymer obtained from the annual agricultural crop *Cyamopsis tetragonolobus*, belonging to family *leguminosae*. As a component of endosperm, Guar gum comprises 35–42% of the dry weight of seed [25]. India, Pakistan, Sudan and the USA are the biggest Guar producing countries. Especially, India grows more than 80% of the world’s Guar, most in Rajasthan state, due to its arid and prevalent monsoon climate, as well as cheap labor [70]. Guar gum has been utilized as emulsifier, thickener, and stabilizer in the food and beverage industry and pharmaceutical chemicals in the pharmaceutical and cosmetics industry for a long time. Nowadays, the petroleum industry is the biggest consumer of Guar gum and more than 40% of the world’s Guar is used as additives in hydrofracking fluid. It is approximated that a traditional hydrofracking project consumes 80 acres’ annual yield of Guar (200–500 kg/acre). The high demand of Guar in the petroleum industry results in a big export volume of Guar from India to the USA. In 2013, around 800 thousand tons of Guar gum was produced globally, out of which 300 thousand tons were exported from India to the USA [70]. As one kind of commodity closely related to oil production activity, the price of Guar gum exhibits big fluctuations corresponding to the oil price. The price of Guar gum was up to USD 20/kg in October 2012, when the oil price was USD 90 per barrel. After that, the price suddenly dropped to less than USD 2/kg in November 2014 with a USD 40 oil. The price of Guar has maintained around USD 2/kg for five years since then, making the culture of Guar not economically attractive outside India.

**Figure 5:**
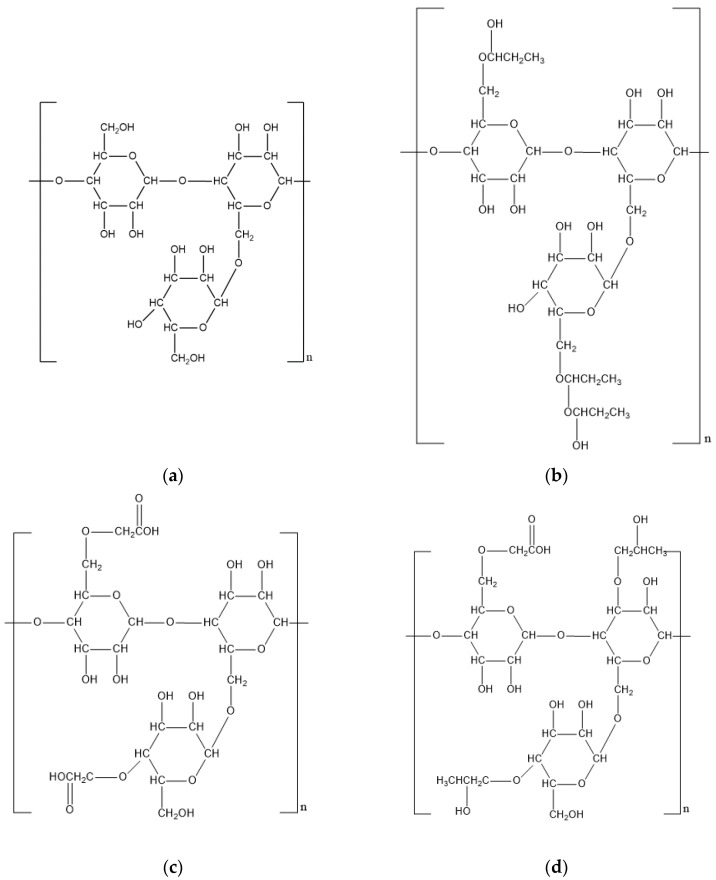
The molecular structure of (**a**) Guar gum; (**b**) Hydroxypropyl Guar; (**c**) Carboxymethyl Guar; (**d**) Carboxymethyl hydroxypropyl Guar.

The Guar gum molecule consists of linear backbone chains of (1–4)-β-d-mannopyranosyl units and the branch points of α-d-galactopyranosyl units attach the backbone by (1–6) linkages, shown in Figure 5a. The average *M*_W_ of Guar gum is in a range of 10^6^ to 2 × 10^6^ Da and the ratio of mannose to galactose units is varied from 1.6:1 to 2:1 [26,27]. The Guar gum solution reveals extremely high viscosity, as 1% of the polymer can increase the viscosity of water to 10,000 mPa·s. Its supreme thickening effect lies on the hydration of galactomannans and the inter-molecular chain entanglements between side chains and backbone [28]. A shear-thinning phenomenon is observed with Guar gum solution and the viscosity is also affected by the pH and temperature: Guar gum solution reaches its highest and lowest viscosity at pH 6–9 and pH 3.5, respectively [29]; the elevation of temperature causes the decrease in viscosity, as the high temperature inhibits the interaction between the water and polymer molecules. The gelation capacity of Guar gum enables it as an essential additive of hydrofracturing fluid and the gelation can be triggered by a wide type of chemicals and the common crosslinking agents include derivatives of methylene-bis-acrylamide, derivatives of ethylene-glycol-di(meth)acrylate, glutaraldehyde, Borate, and chemicals contain Ti^4+^, Zr^4+^ and Al^3+^ ions. During the gelation, the Guar gum molecules reveal a closed loop-like structure and bonds between the crosslinkers and the hydroxyl groups of polymer chains are formed. The cross-linked network captures the free water molecules in the solution, increasing the water absorption and holding capacity of the hydrogel system [30].

Guar gum is directly extracted from the plant material, making it cheaper than polysaccharide biopolymers obtained from the fermentation process. Sabahelkheir et al. have summarized the prevail protocol as follows [71]: First, the harvested Guar seed is dried, and heated at 100 °C for 30 min to deactivate the enzymes which catalyze the hydrolysis Guar gum during extraction. Then, the endosperm of seed is separated from the hull and embryo, followed by multistage grinding and sieving operations. The powdered Guar gum comprises 75–86% water-soluble galactomannan, 8–14% moisture, 5–6% protein, 2–3% fiber and 0.5–1% ash [72], and additional separation treatments may be required to further purify the gum as the impurity will cause the formation damages in hydrofracking process.

A range of chemical derivatives of Guar gum has been synthesized [73], and the modifications improve the solubility, rheology properties and reduce the number of undissolved residues of polymer in hydrofracking fluid after the breaking process. Hydroxypropyl Guar (HPG), Carboxymethyl Guar (CMG) and Carboxymethyl hydroxypropyl Guar (CMHPG) are three types of derivatives comprehensively studied and evaluated in the petroleum industry. HPG is synthesized in isopropyl alcohol with Guar gum and propylene oxide as substrates, and the process is catalyzed with sodium hydroxide at 60 °C [31]. The carboxymethylation of Guar gum is employed with two-step reaction proceeding with a strong base (sodium hydroxide) that deprotonates the free hydroxyl groups in Guar gum to form alkoxides, then carboxymethyl groups are formed in a reaction with Guar alkoxides and chloroacetic acid [32]. CMHPG is generated with a two-step process that involves both the carboxymethylation and hydroxypropylation reactions, which was described by Pasha and Ngn [33]. The introduction of the hydroxypropyl and carboxymethyl group brings negative charges to the polymer, increasing its hydration rate and thermo-stability. According to their study, CMHPG can maintain its viscosity at temperatures up to 60 °C under a high salinity environment, compared with a 90% viscosity loss of natural Guar gum under the same condition. The modification also changes the crosslinking behavior of Guar gum, and Lei et al. figured out the order of critical crosslinking concentration was CMG< CMHPG< Guar< HPG [34].

### 2.4. Cellulose

Cellulose is the most abundant biopolymer derived from biomass, as the form of lignocellulose of plants. For example, cotton fiber and wood contain 90–95% and 40–50% cellulose, respectively. It is physically and chemically bonded with lignin and hemicelluloses, contributing to the shape and structure of plant cells. Besides extracting from plants, cellulose can also be obtained through the fermentation of bacterial species such as *Acetobacter Xylinam*, and the yield is between 5–15 g/L [35]. The *M*_W_ and dispersion index of microbial cellulose are more homogenous than plant-originated cellulose, making them a better material for pharmaceutical applications. When cellulose was first isolated from plant by Anselme Payen in 1839, people were surprised that cellulose and starch exhibited the same molecular formula as (C_6_H_10_O_5_)_n_, while obvious differences in the solubility and textile between them were recognized. Since then, intensive research has been done to investigate the molecular conformation of cellulose and the links between the conformation and its chemical and physical properties. Cellulose and its derivatives are widely utilized to manufacture paper and fibers. Moreover, the high degree of crystallinity and polymerization, as well as the high specific surface area of cellulose makes it an indispensable material for the fine chemistry industry. It is estimated that the annual yield of cellulose is between 10^10^–10^11^ tons around the world, and about 6 × 10^9^ tons are utilized in the pulp, textile, materials and chemical industries [74]. There is a growing interest in the application of cellulose, especially microcrystalline cellulose (purified and partially depolymerized cellulose), as a thickening agent for EOR, filtration control agent for drilling and wastewater treatment sections in the petroleum industry.

**Figure 6:**
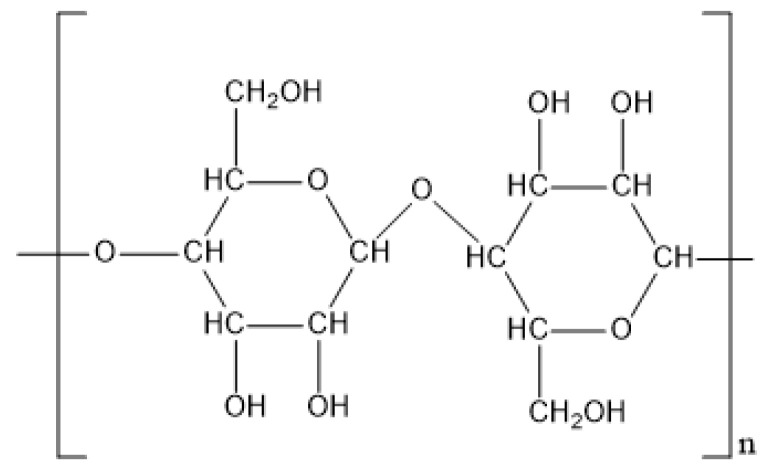
The molecular structure of cellulose.

The *M*_W_ of native cellulose is around 2 × 10^6^ Da with the degree of polymerization approximately 10,000 [36]. It consists of d-glucopyranose ring units linked in a (1–4)-β fashion with the chair configuration and three hydroxyl groups exist in each anhydroglucose unit, bringing degradability and chemical variability (Figure 6). Cellulose exhibits a crystalline fiber structure due to the hydrogen bonds between the hydroxyl groups on one chain and the oxygen atoms on the same or on a neighbor chain [37]. The hydrogen bonds cause a limited solubility of cellulose in most solvents and the tendency of self-aggregation. Interestingly, the location of hydrogen bonds between and within strands is different between plant originated (Iα), microbial (Iβ) and regenerated cellulose (II), leading to different stability. The hydrophobic areas around the carbon atoms of cellulose, as well as the high surface weight ratio (400–900 m^2^/g), bring an excellent adsorption capacity of oily contamination [38].

**Figure 7:**
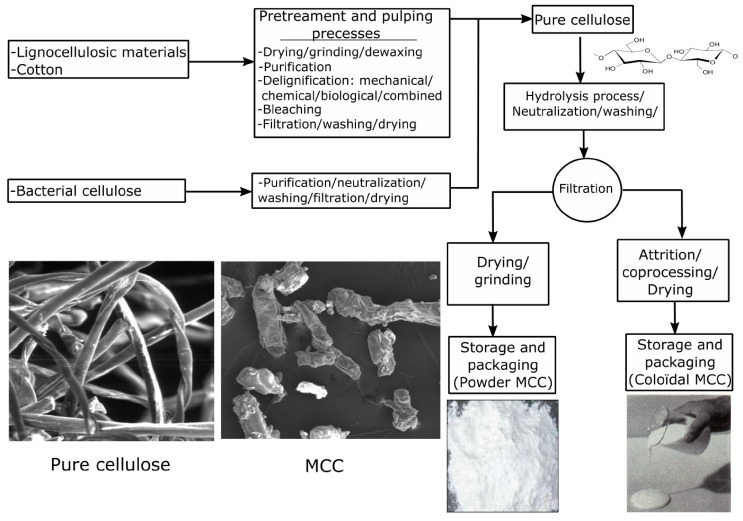
The manufacture process of microcrystalline cellulose, reproduced with permission from [77]; copyright Elsevier, 2016.

The commercial cellulose in the microcrystalline structure and the manufacturing process was shown in Figure 7. First, pure cellulose is separated from plants with physical or chemical processes [56]. Then, acid hydrolysis of cellulose is conducted with 1–2.5 M HCl or H_2_SO_4_ solution at 80–105 °C aiming to selectively remove the amorphous region of cellulose fiber, but retain the microcrystalline region [75]. Finally, the microcrystalline cellulose (MCC) is neutralized, washed and spray-dried to generate the product with various particle size distribution, moisture content, and binding ability [76]. The average price of microcrystalline cellulose on the market is around USD 4/kg, and the utilization of cellulose is considered feasible due to its abundance and economics.

To meet the requirements of solubility, rheology in the petroleum industry, chemical modification of cellulose is a prevailing approach. Hydroxyethyl cellulose (HEC), Carboxymethyl cellulose (CMC) and amphoteric cellulose are common cellulose derivatives. HEC is a nonionic soluble cellulose derivative and it can easily dissolve in either hot or cold water and produce solutions with a wide range of viscosities. To synthesize HEC, pure cellulose is treated with a sodium hydroxide solution to swell the cellulose and form active alkali cellulose. Then, ethylene oxide is added to trigger a series of etherification reactions to form HEC [39]. Carboxymethylation is a chemical approach to introducing carboxyl groups on the surface of cellulose and the medication renders the cellulose soluble and chemically reactive. Moreover, CMC is also recognized as an inexpensive, nontoxic, highly biocompatible and biodegradable material. To make it, cellulose, sodium hydroxide and urea are mixed and stirred continuously until a slurry mixture appears. Then the mixture is cooled in a refrigerator to −12.5 °C, followed by being stirred vigorously at ambient temperature to obtain the transparent cellulose solution. The carboxymethylation of cellulose is triggered by adding sodium monochloroacetate in the cellulose solution with vigorous stirring at 55 °C for 5 h. Finally, CMC is precipitated with methanol and neutralized with dilute acetic acid [40]. Amphoteric cellulose contains both anionic and cationic groups, leading to a remarkable solubility across the entire pH range and flocculation effect [41]. Amphoteric cellulose can be synthesized by adding 3-chloro-2-hydroxyl-propyltrimethyl ammonium chloride and 3-chloro-2-hydroxypropanesulfonic acid sodium salt into the cellulose solution orderly with a 2500 rpm at 25–45 °C [42], and the degree of substitution values for positively and negatively charged groups can be controlled by manipulating the reaction rate and dosages of cationic and anionic reagents.

### 2.5. Chitin and Chitosan

Chitin is a ubiquitous natural polysaccharide that exists in the shells of crustaceans, exoskeletons of insects, and cell walls of fungi, providing the strength for the structures. Most of the commercial Chitin comes from crustacean shells, a cheap and abundant byproduct in the food industry and it comprises up to 30% of the dry weight of shells. As the shells also contain a high-value pigment called carotenoids, the integration of the production of Chitin and carotenoids guarantees the profits of the business. Exhibiting the similar molecular structure, Chitosan is the partially deacetylated derivative of Chitin obtained via the alkaline treatment. Both Chitin and Chitosan are biodegradable with low toxicity and they are widely used in the biomedical and pharmaceutical industry as an ideal material for immobilizing enzyme, creating affinity chromatography column, manufacturing wound-dressing material, controlling drug release, etc. [43]. In the petroleum industry, there is increasing interest in applying Chitin and Chitosan in the management of oily water and industrial pollutants, regarding their high adsorption capacity [44]. Due to the increasing demand for Chitin, the global market size of Chitin/Chitosan reached USD 2 billion in 2016 with a 15–20% annual growth rate. Japan and the USA are the two biggest producers which collectively comprise two-thirds of the global yield.

**Figure 8:**
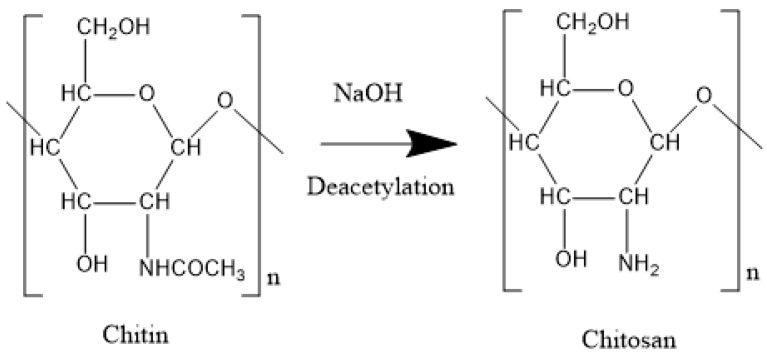
The scheme of conversion of Chitin to Chitosan.

Chitin is composed of β (1–4)-linked 2-acetamido-2-deoxy-β- d-glucose (N-acetylglucosamine) and it is converted to Chitosan when more than half of the d-glucosamine is N-deacetylated (Figure 8) with an increase in solubility in acidic solution. The *M*_W_ of Chitosan is in the range of 2 × 10^3^ to 10^6^ Da, and the wide range brings versatile applications. For example, Tsaih and Chen observed that Chitosan with *M*_W_ less than 8.6 kDa had a better aggregation effect and lower gelation temperature in the pharmaceutical application [45], as the Chitosan molecules possessed a more rigid and extended stiffness [46,47]. To obtain the required texture, the gelation of Chitosan can be triggered with both physical and chemical cross-linking agents. Physical cross-links are formed with weak interactions such as hydrogen and ionic bonds and the prevailing physical crosslinkers include citric acid, dextran sulfate and phosphoric acid. Compared with physical crosslinks, chemical crosslinks lie on the covalent bonds with stronger interaction, and chemicals such as glutaraldehyde, formaldehyde, tripolyphosphate and polyaspartic acid sodium salt are all common crosslinking agents [48]. Chitosan is widely used in the petroleum industry for wastewater treatment through the charge neutralization mechanism. According to this mechanism, Chitosan possesses high charge density due to the amine group of its structure, and these charged sites can bind anionic substrate on its surface, causing the destabilization of the colloids of waste oil and emulsion [49].

The production of Chitin mainly involves the removal of proteins and calcium carbonate from shells of crustaceans. To realize it, the shells are first reacted with NaOH or KOH at 95 °C for 16 to 48 h to dissolve the protein, followed by HCl treatment for 1 to 24 h to remove the calcium carbonate [78]. The isolated Chitin can be further deacetylated in 40% NaOH at 120 °C for 1 to 3 h to generate the Chitosan with 70% deacetylation. The market price of Chitosan is around USD 220/kg, much higher than cellulose and Xanthan gum due to its time-consuming and expensive purification process [50].

Modifying the *M*_W_ of Chitosan is a prevailing approach to improving its rheology, gelation and aggregation property, and the decrease in *M*_W_ can be achieved through chemical, enzymatic and mechanic methods [51]. For the chemical method, Chitosan weathers chemical degradation with acid or alkali treatment at high temperature. Although the chemical approach is suitable for industrial-scale production due to its high reaction intensity, precisely controlling the *M*_W_ is difficult and the product exhibits a wide distribution of *M*_W_ [52]. The enzymatic degradation is realized by hydrolyzing Chitin/Chitosan with Chitinase/Chitosanase. The high reaction selectivity is helpful to maintain the integral structure of the product, resulting in a more homogeneous distribution of *M*_W_. However, the enzymatic treatment is only feasible for the production in lab-scale due to its high cost [53]. The principle of mechanical methods lies in the physical force to break the chemical bonds between units of Chitin or Chitosan and the physical force can be generated from shearing, ultrasonication, and micro fluidization flow [54,55,56]. The mechanical method is environmentally friendly, energy effective, and effects are focused on increasing its process rate.

## 3. Evaluation of Biopolymer

Before utilizing biopolymers as various fluid additives in the oil field, the evaluation of their performance is the necessary step to ensure the safety and efficiency of the treatment. The common interesting characteristics of polymer in the petroleum recovery include viscosity, filterability, adsorption, gelation, and stability. Since these characteristics are strongly affected by the working condition, the evaluations are conducted in a lab with the parameters, such as flow rate, temperature, pressure, etc., carefully controlled to mimic the working condition.

### 3.1. Rheological Analysis

The rheology properties of fluids with additives are commonly evaluated with various rotational viscometers and rheometers. During the analysis, the torques necessary to rotate the fluid with prepaid angular velocities were recorded to reveal the relationship between the shear stress and shear rate, based on the known dimension parameters. In practice, several mathematical models have been developed to describe the shear stress/shear rate relationship as described below:

Bingham Plastic Model:

(2) τ=YP+PVγ

where *τ* is shear stress; *γ* is shear rate; *YP* is yield point (stress); *PV* is plastic viscosity.

Power Law Model:

(3) $$\tau =K\gamma^{n}$$

where K is consistent index; *n* is flow behavior index.

Herschel–Bulkley Model:

(4) $$\tau =\tau_{0}+K\gamma^{n}$$

where $\tau_{0}$ is the yield stress.

Gasson Model:

(5) $$\sqrt{\tau }=\tau_{0}+\mu_{p}\sqrt{\gamma }$$

where $\mu_{p}\;$ is Casson plastic viscosity coefficient.

As two-parameters models, Bingham Plastic and Power Law Models are applied as simple yet useful tools for viscosity description in the petroleum industry of the past half-century. According to Equation (2), the shear stress of fluids exhibits a linear dependence on shear rate as it is beyond the yield point in the Bingham Plastic Model. In contrast, the Power Law Model neglects the yield point and reveals a non-linear behavior of shear stress with rate, which is preferred in the low-shear-rate condition. These facile models, however, only provide a rough description of the viscosity profile. To achieve a better delineation of the viscosity profile in petroleum recovery, the research community has established more complex models such as Herschel–Bulkley and Gasson models, accommodating both the existence of the yield point and the non-linearity relationship [79].

### 3.2. Filtration Test

The filtration test is specially designed to obtain the filtration property of polymer solutions regarding the reservoir formation, which is a critical parameter of drilling and polymer flooding fluids. In the drilling process, the invasion of drilling fluid into the formation is unwanted as it can cause formation damage, as well as increase the drilling cost. In contrast, the flow of polymer solution through the formation is necessary for the polymer flooding process. In the filtration test, the polymer solution is filtered through filter paper at constant pressure and the filtered volume versus time is measured [80]. Alternately, the pressure drop across the filter paper versus filtered volume is recorded with a constant filtration rate [81]. Any increased pressure drops, or decreased filtration rates, indicate the plugging of the filter and removal of components from the polymer solutions and the flow rate through the cake can be expressed as:

(6) $$\frac{dV_{f}}{dt}=\frac{kA∆p}{\mu h_{c}}$$

where $V_{f}$ t, k, A, ∆p, μ, *hc* are the filtrate volume, time, permeability, cross-section area, pressure drop across the filter cake, viscosity of solution and thickness of filter cake, respectively.

### 3.3. Surfactant–Polymer Compatibility Test

To achieve a higher recovery rate, surfactant–polymer flooding or alkaline–surfactant–polymer flooding is widely applied when polymer and surfactant co-exist in the aqueous phase. In that case, the surfactant–polymer compatibility needs to be tested to deliver the efficiency of flooding. The test is typically conducted in glass test tubes with an aqueous solution of surfactant, and temperature, pH, as well as the salinity, are carefully controlled at the prepared levels. The compatibility can be exhibited if the aqueous solution remains clear and stable when the polymer is added to the solution. In contrast, if the polymer causes a three-phase microemulsion to form a separate, polymer-rich aqueous phase, the polymer and surfactant are considered as incompatible. In practice, co-solvents may be added to improve the surfactant–polymer compatibility.

### 3.4. Core Flooding

**Figure 9:**
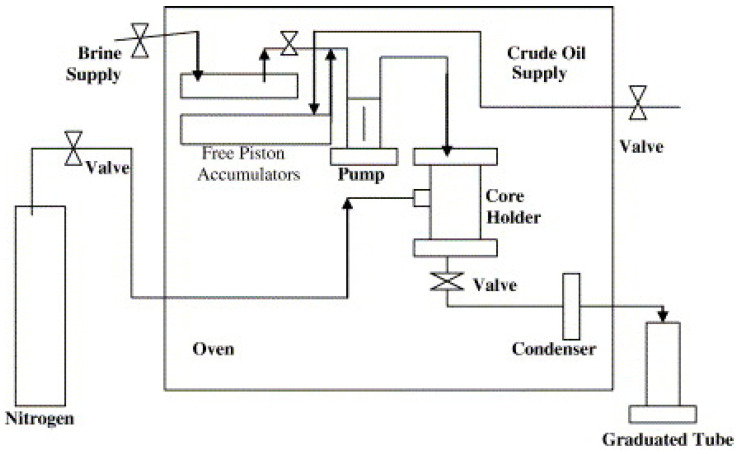
The scheme of a traditional core flooding experiment, reproduced with permission from [82]; copyright Elsevier, 2006.

Core flooding is a laboratory test that involves placing a core sample in a pressured cell and injecting a fluid or a combination of fluids through the sample, aiming at evaluating the performance of fluid flooding (Figure 9). The conditions may be either ambient temperature and low confining pressure or high temperature and pressure of a subject reservoir. During the experiment, the core sample is initially saturated with a combination of brine and oil to mimic the initial oil saturation condition. Then the fluid is injected through the sample with a prepared flow rate or pressure. By monitoring the pressures and flow rates at both ends of the core, the permeability of core and the replacement rate of the fluid can be determined. The formation damage caused by the fluid injection, or interactions between the fluid and the rock can also be investigated after the test or be simultaneously revealed during the test by other measurements such as nuclear magnetic resonance (NMR).

## 4. Application of Polysaccharide Biopolymer in Petroleum Recovery

The petroleum recovery process is composed of many operations including drilling, hydraulic fracture, production, plugging, and treatment of wastewater. During these operations, biopolymer additives are added to meet the special requirement of fluids’ rheology, compatibility, and stability. As the reserve of conventional reservoir decreases annually, more concerns have been attracted to exploit and extract petroleum from unconventional reservoirs, such as shale and heavy oil. In 2019, 7.7 million barrels per day of shale oil were produced from shale oil resources in the United States, which equals 63% of total national crude oil production. Generally speaking, the oil production from unconventional reservoirs requires more participation of (bio)polymers due to its complicated extraction process and the increasing activity related to unconventional resources stimulates the study of biopolymers in the petroleum industry.

### 4.1. Drilling Fluid

**Figure 10:**
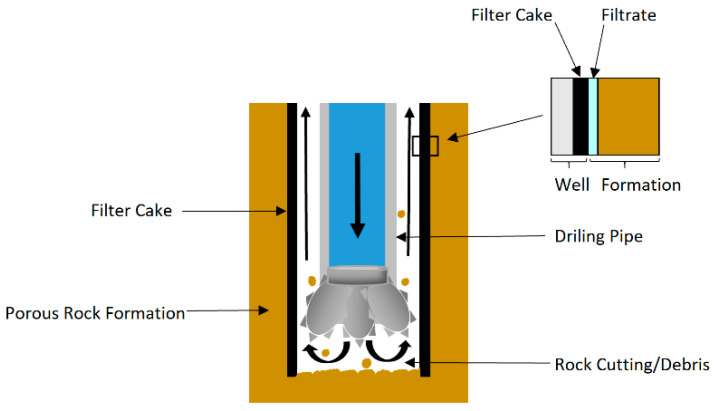
The scheme of drilling fluid circulation during the drilling process.

At the first stage of the petroleum recovery, drilling means generating a pathway between the surface to the petroleum reservoir by fracturing the rock. During the process, drilling fluid (mud) is used to deliver the fractured debris from the drilling bite to the surface, and the mud also plays a role in the stabilization of borehole walls by maintaining the hydrostatic pressure and sealing the well wall to reduce the fluid loss to the formation, shown in Figure 10. Based on the composition, drilling fluid can be classified as water-based and oil-based fluids, and the former is preferred by the industry due to its low cost and environmental impact. A traditional water-based drill fluid contains clay (gel for sealing), barite (weight material) and (bio)polymers with various functions. For example, Xanthan gum is added to improve the rheological properties of drilling fluids; polyanionic cellulose (PAC), Carboxymethyl cellulose (CMC) and starch are utilized as fluid loss control agents. The performance of drilling fluid can be evaluated based on its rheological properties, fluid loss prevention capacity and stability.

#### 4.1.1. Rheological Properties of Drilling Fluid

Viscosity is a critical parameter of drilling fluid to deliver the drilling cutting and clean the drilling hole. A common drilling fluid has a viscosity between 5 mPa·s and 25 mPa·s, and more viscous fluid is required for a deep drilling well. In practice, adding (bio)polymer is a prevailing approach to increasing the viscosity of drilling fluid and the Herschel–Bulkley and Gasson models are preferred in the delineation of viscosity profile of drilling fluid, as they accommodate both the existence of the yield point and the non-linearity relationship [79]. Table 3 summarized examples of rheology properties of drilling fluids with biopolymer. As shown in Table 3, the yield stress ($\tau_{0}$) of fluid is commonly adjusted between 1 and 10 Pa, which needs to be high enough to carry cuttings, but not too high to generate pump pressure for starting mudflow. The consistent index (K) and Casson plastic viscosity coefficient ($\mu_{p})$, both related to the shear-thinning or thickening effect, were also carefully controlled to prevent the malfunction of drilling fluids with an inconstant shear rate.

As an economic and feasible biopolymer, Xanthan gum has been widely used as an additive in drilling fluid since the 1930s. Its high *M*_W_, strong bonding between the chain, and elastic structure bring an excellent thickening effect and transportation capacity. By now, Xanthan gum has become the most prevailing bio-thickening agent in drilling fluid [89]. Scleroglucan exhibits a similar thickening effect and better stability at high salinity conditions, which make it preferred in harsh drilling environments [83]. Besides refined biopolymer, various biopolymers in the form of raw material were also used in drilling fluid. For example, SaharKafashi et al. succeeded in applying bagasse as one kind of economic and feasible biopolymer, Xanthan gum has been widely used as an additive in drilling fluid since the 1930s. Its high *M*_W_, strong bonding between the chain, and elastic structure bring an excellent thickening effect and transportation capacity. By now, Xanthan gum has become the most prevailing bio-thickening agent in drilling fluid [89]. Scleroglucan exhibits a similar thickening effect and better stability at high salinity conditions, which make it preferred in harsh drilling environments [83]. Besides refined biopolymer, various biopolymers in the form of raw material were also used in drilling fluid. For example, SaharKafashi et al. succeeded in applying bagasse as a thickening agent in drilling fluid to increase the viscosity of fluid almost two times [90]. Taiwo also invented a recipe of drilling fluid with cassava starches, substituting the imported expensive thickening agent, aiming to reduce the cost [91]. The thickening effect of biopolymer is greatly influenced by environmental parameters including temperature, pressure, ionic concentration and pH, as well as the interactions between polymers and other components of drilling fluid such as clay, salt, bactericide, shale inhibitor, etc. [83,92,93,94]. For instance, the existence of weighting material significantly increases the yield stress and consistent index of fluid due to the aggregation between polymer and weighting material [95], and the influence was varied based on the category of added polymer [83].

#### 4.1.2. Fluid Loss Prevention

During the drilling process, the fluid loss occurs when fluid is invading into the formation near the drilling well. The fluid loss is unfavorable as it increases the process cost and causes the potential formation damage. To minimize the fluid loss, drilling fluid is expected to exhibit high viscosity and the capacity to form a thick filter cake with low permeability. To address this concern, biopolymer can be helpful through four basic mechanisms that affect filtration including bridging, bonding, deflocculation and viscosity [96]. Although Xanthan gum can increase the fluid viscosity, the utilization of it as a filtration control agent usually failed because its chains can easily pass through the small pores of the formation, instead of forming thick filtering cake [89]. Moreover, the later research proved that Xanthan gum can interact with the swollen clay of the drilling fluid, resulting in a porosity and permeability increase in filtration cake, and therefore increase the fluid loss [87]. Instead of Xanthan gum, polyanionic cellulose (PAC), especially Carboxymethylcellulose (CMC), is a common filtration control agent in drilling fluid. The negative molecular chain of CMC attaches the positively charged edges of clay platelets. Therefore, CMC extends like fingers into the cake pores, increasing the compactness and the stability of the filter cake against electrolytes disturbing and temperature aging [97]. Based on the results of the filtration test, a 50% reduction in fluid loss can be achieved with 10 g/L PAC in drilling fluid. Moreover, the improvement can be further enhanced by tailoring the size distributions of PAC: reducing the size diameter from 3000 nm to 91 nm, Fereydouni et al. realized an additional 12% reduction in fluid loss [98]. Song also indicated that small and compacted polymer structures were more favorable for forming ideal filter cakes with low permeability, based on his study on the nanofiber and nanocrystal of cellulose [85]. The capacity of raw agriculture residue such as date, grass, and grass-ash, soybean isolation, rice husk, etc. as fluid loss agents are also widely mentioned in recent studies. For example, Li et al. added soybean isolation (6 wt%) into drilling fluid and reduced the filtration rate from 7.5 mL/s to 2.5 mL/s due to the permeability decrease in filter cake [99]. Through another mechanism, the function of rice husk as fluid loss agents lies in its ability to increase the thickness of filter cake [100].

#### 4.1.3. Drilling Fluid Stability

The stability of drilling fluid is considered as another important factor to determine the efficiency and safety of the drilling process, especially when drilling at high temperature or extreme pH is required. At high temperature, biopolymers will weather a degradation through the thermo-oxidation mechanism and the degradation can be accelerated with a high concentration of hydroxide. As a result, biopolymers are not recommended as drilling fluid additive when the temperature of wellbore is higher than 100 °C and the pH is higher than 8 [101]. Aiming to increase the application scope of the biopolymer in the drilling section, recent research focuses on screening chemical additives that help the biopolymer against harsh environments. According to the research conducted by Zou et al., collectively applying crosslinkers and deoxidants can improve the thermostability of Xanthan gum. The former can limit the conformation change of polymer molecules caused by heating, and the latter can prevent unwanted oxidation-reduction reactions. After adding borax and sodium sulfite, Xanthan gum solution can retain 40% of its viscosity at 120 °C, compared with a total loss of viscosity without treatment [102]. Formate salts, another kind of deoxidants, were also applied to inhibit the thermal degradation of biopolymer up to 150 °C [103]. The improvement of thermostability was also achieved with additional polyethylene glycol as a sacrificial scavenger, which moped up free radicals before they could attack the biopolymer [104].

### 4.2. Hydraulic Fluid

To deal with low and ultra-low permeability reservoir formations, such as shale, hydraulic fracturing along the horizontally drilled well is a well-established practice to improve the productivity. By injecting the hydraulic fluid into the formation with high pressure (30 to 200 atm) and flow rate (200 L/min), hydraulic fractures can be triggered and propagated, leading to a superior inflow performance of the well mainly due to the increase in the cross-sectional area of fluid motion. The first hydrofracturing process was conducted by Clark in the 1940s [105], and the stress on petroleum recovery from shale in this century provides a continuous driving force for the quick development of this process. To ensure a successful and efficient hydraulic fracturing operation, selection and screening of the ideal hydraulic fluid are critical. At the early stage, oil-based fluids such as kerosene, crude oil, or gasoline were used as hydraulic fluid and fatty acids were added to increase the fluid viscosity [106]. Later, water-based fluids with biopolymer as thickening agent have replaced oil-based fluids and have become the preferred choice for most hydraulic operations due to the concerns of process safety and environmental regulations. Based on the various status of hydraulic fluid, the hydrofracturing process can be divided into three stages, and the viscosity of fluid needs to be carefully controlled to meet the different requirements on each stage.

The first stage: hydraulic fluid is pumped into the downhole with high pressure to trigger and propagate the fractures, aiming to increase the drainage volume of the reservoir. However, the increased drainage volume also causes a significant amount of hydraulic fluid leakage into the neighboring formation. To minimize it, a linear polymer is added into the fluid to increase the viscosity up to 100 mPa·s.

The second stage: after the propagation, a cross-linker is pumped and mixed with hydraulic fluid containing the linear gel, resulting in a significant increase in the viscosity of hydraulic fluid up to 1000 mPa·s. Such high viscosity is critical to the transportation and distribution of the proppants, small and solid particles designed to keep the fracture open, through the fracture.

The third stage: at the end of hydraulic fracturing process, the hydraulic fluid quickly leaks to the formation, releasing the pressure and allowing hydrocarbon to flow into the fractures. To realize it, the viscosity of hydraulic fluid needs to be decreased by injecting breaker solutions. The breaker can cause the degradation of polymer, which is essential as the remaining polymer may cause the permeability damage of the formation.

To meet the requirements above, the ideal biopolymer additive must access versatile properties including a proper viscosity, ability of quick and reversible crosslinking, and feasibility of controlled degradation. In the petroleum industry, Guar gum, as well as its derivates such as Carboxymethyl hydroxypropyl Guar (CMHPG), dominate the market due to their excellent thickening effect and low cost.

#### 4.2.1. Rheology Properties of Linear Biopolymer

The viscosity of hydraulic fluid containing linear biopolymer is affected by various parameters including polymer category and concentration, shear rate, environment temperature, salinity, and pH. While controlling the environmental factors is limited in the petroleum recovery, the manipulation of polymer concentration can still provide enough room for obtaining the targeted viscosity [107]. Table 4 summarizes examples of the viscosity profile of biopolymer under various condition.

As shown in Table 4, a higher content of biopolymer commonly results in a more viscous fluid, and the prevailing working concentration of polymer is between 0.1 and 1 wt% in hydraulic fluid. For Guar gum solution, one order difference of viscosity, from 10 mPa·s to 103 mPa·s, can be achieved by elevating the content of polymer from 0.24% to 0.95 wt%. The utilization of Guar gum on high centration may confront a dissolving difficulty due to the insoluble poly-mannose backbone of Guar. Unwanted fisheyes, agglomerates of partially hydrated powders, may be observed in the injection well when the Guar gum concentration is higher than 1 wt% [112]. While the Guar gum solution reveals some kind of resistance against the effect of salinity fluctuation on viscosity, its thickening effect was still reported to be inhibited at extremely high salinity conditions [109].

Besides polymer content, injection rate and temperature also determine the recipe of a biopolymer solution. Compared with synthetic polymers, the poor thermostability is the most disadvantaged in Guar gum, which makes it unsuitable at higher temperatures than 83 °C. Holding better thermostability, modified Guar such as Carboxymethyl Guar gum (CMG), Carboxymethyl Hydroxypropyl Guar (CMHPG) and cellulose-based polymers such as Carboxymethyl cellulose (CMC), Hydroxyethyl cellulose (HEC), and Carboxymethyl hydroxyethyl cellulose (CMHEC) are alternative biopolymers applied at high temperature [113,114]. The modification brought excellent stability that maintains the viscosity at temperatures up to 160 °C [115]. However, due to the higher cost yet worse thickening effect, utilization of these alternatives is not as economic as native Guar gum. In some cases, Guar gum and synthetic polymer are collectively utilized to improve the performance of hydraulic fluid at high temperature and shear rate [111,116].

#### 4.2.2. Biopolymer Crosslinking

To improve the capacity of transporting proppants, a crosslinker is added to react with cis-OH pairs on the galactose side chains of Guar gum, resulting in a dramatic increase in the viscosity. Borate, Ti^4+^, Zr^4+^, and Al^3+^ ions are all common crosslinkers of Guar gum solution, and their application scopes are restricted by pH, temperature, as shown in Table 5.

The borate can react with the cis–OH groups of Guar gum to form inter-and intra-molecular crosslinking with hydrogen and ionic bonds. As the degree of crosslinking is controlled by the reaction equilibrium, the higher concentration of borate is required at low temperatures and high pH [117]. The working concentration of Borate is around 0.024–0.09 wt%, and it is not recommended at high temperatures due to its stability. Different from Borate, the variety of organic ligands or chelating agents containing Ti^4+^ and Zr^4+^ ions can trigger crosslinking with covalent bonding, which is more favorable at high temperatures. At low pH, Al^3+^ ion is more effective than Borate and it is preferred in acid fracturing application where CO_2_ compatibility is needed. However, metal crosslinkers may cause formation damage and loss of fracture conductivity, which make them not as popular as borate [118]. Recent research is focusing on designing novel crosslinkers with better stability and efficiency. For example, Sun and Qu have invented the new borate crosslinkers including thiophenediboronic acid, benzenediboronic acid, and biphenyldiboronic acid. Compared with Borax, the novel crosslinkers can bring a higher viscosity due to their bigger molecule size [119]. Geetanjali Chauhan also found a novel Zr-Karaya gum crosslinked gel, which can maintain the stability up to 150 °C with fewer polymer residues [120]. In some cases, the delay of crosslinking is necessary to reduce friction during the delivery. To realize it, Legemah et al. succeeded in chelating Zr^4+^ complexes with a mixture of alcohol, carboxylic acid and amine functional groups [121]. Kalgaonkar and Patil also reported that increasing the pH with adding carbonate can cause a delay of Zr^4+^ crosslinked gels [122].

#### 4.2.3. Biopolymer Breaking

In the last stage of the hydraulic process, breaker is added and reacts with the polymer, reducing the viscosity of fluid and enabling the hydrocarbon flow. Based on the mechanism, the breaker can be categorized as enzymes and oxidizers, as shown in Table 6.

The oxidizer defragments the polymer into shorter molecules through generating radicals from the decomposition of persulfates, which is more effective at high temperatures. However, the generated radicals can cause damage to the pump equipment. Compared with oxidizers, enzyme exhibits better substrate-selectivity. The hemicelluloses can specifically cleave the ether bonds in the mannose backbone, without the influence of the process equipment. Moreover, as one kind of catalysis, the enzyme itself will not be consumed during the breaking process, bringing extremely high efficiency at low cost. However, the enzyme requires a proper environment to achieve full activity and it can totally lose its activity when the temperature is higher than 135 °C or pH is higher than 10.5. Screening enzymes with higher thermo-stability [123] and immobilizing enzymes with nanoparticles to help against harming the environment [124] are promising approaches to enlarging the application of enzyme as breakers. After the breaking treatment, some residues of polymer retain in the fracture, causing formation damage. Regarding that, the enzyme is preferred to oxidizer as it can provide a more homogeneous breaking, leaving less residue [125]. Using modified gums, such as CMHPG and HPG, are another way to prevent formation damage as they only leave 2–4% of residues, much fewer than native Guar gum (6–10%).

### 4.3. Enhance Oil Recovery

**Figure 11:**
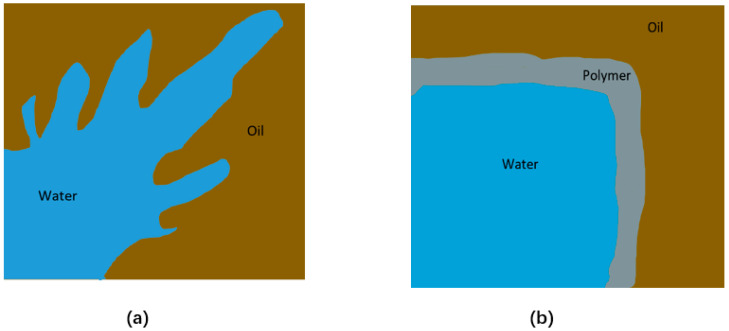
The effect of viscous fingering on water flooding: (**a**) unfavorable mobility ratio; (**b**) favorable mobility ratio with polymer treatment.

The pressure of the reservoir decreases gradually with the progress of oil production and finally fails to drive more oil to the production well. To continue the production, water is injected into the formation from the injection well, building up the pressure of the reservoir and maintaining the oil flow. However, the performance of water flooding is deleteriously affected by viscous fingering, which happens with an unstable displacement of a more viscous fluid by a less viscous fluid (shown in Figure 11). The severity of viscous fingering is highly related to the mobility ratio (*M*), which is given by:

(7) $$M=\;\frac{k_{w}/\mu_{w}}{k_{o}/\mu_{o}}$$

where $k_{w}$ and $k_{o}$ are the permeability of water and oil, respectively; $\mu_{w}$ and $\mu_{o}$ are the viscosity of water and oil, respectively.

**Figure 12:**
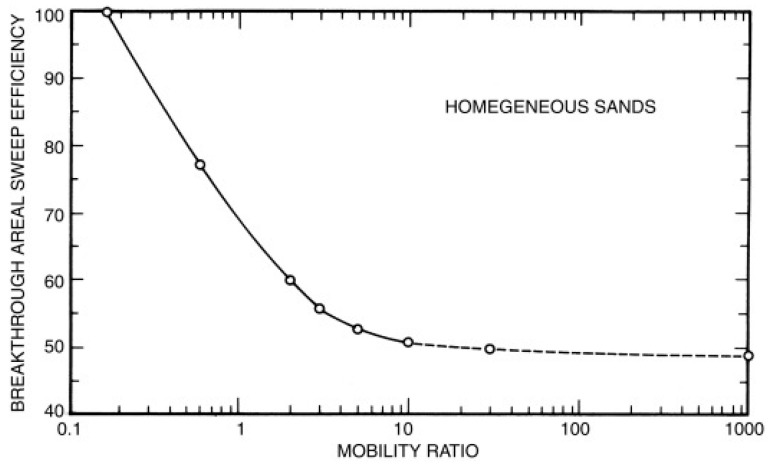
The effect of mobility ratio on areal sweep efficiency at breakthrough.

According to empirical data, the injected water tends to bypass oil and an early breakthrough is expected when the mobility ratio is greater than one, resulting in an unfavorable water flooding. The correlation between mobility ratio and area sweep efficiency, the fraction of the pattern area from which the reservoir fluid is displaced by the injected phase at the time of breakthrough, is shown in Figure 12.

In most water flooding cases, the mobility ratio should have a value equal to or less than 10 and it can be reduced by increasing the water viscosity by adding polymers. The early pioneering work on polymer flooding can be tracked as early as 1960 with Pye and Sandiford [126,127]. The first large commercial application of polymer flooding was conducted in the United States in the 1970s, but the number of projects abruptly decreased in the 1980s due to the low oil prices. Interestingly, the polymer flooding has regained the attention of the oil fields around the world, especially China, since the 1990s. For example, the polymer flooding in the Shengli and Daqing oilfields of China increased the oil recovery rate by 6 to 12%, contributing to 250,000 barrels per day in 2004 [128].

As the polymer flooding may continue for years, the long-time interaction between the polymer and reservoir makes the screening and optimizing of polymer recipe extremely critical and complicated. To achieve successful polymer flooding, the operator faces a series of challenges such as polymer viscosity manipulation, formation damage, flooding compatibility, and polymer stability.

#### 4.3.1. Rheology Properties of Polymer Flooding Solution

The most imperative property of polymer solution is its ability to generate viscosity at a minimum concentration. Typical polymers for water flooding include synthetic polymers such as partially hydrolyzed polyacrylamide (HPAM), and various biopolymer such as Xanthan gum, Scleroglucan, cellulose and Carboxymethyl cellulose (CMC) and their rheology parameters of the power–law model were shown in Table 7.

The biopolymers exhibit the same order of thickening effect as HPAM and the desired viscosity can be achieved by applying various concentrations. Among them, Xanthan gum possesses the highest thickening effect, making it the preferred polymer for the operation. Interestingly, all the polymers hold a shear-thinning behavior [132,133,134] as the apparent viscosity decreases with the elevated shear rate, and the severity of the behavior defines the flow behavior index (n). Actually, the shear-thinning behavior is favored in polymer flooding. When the polymer is injected through the well, the high shear rate thins the viscosity, reducing the pressure for pumping. In contrast, when the polymer solution contacts the formation, the decreased shear rate results in higher viscosity, benefiting the oil displacement. Besides allowing an increase in the viscosity, the ideal biopolymer should also be in abundance, feasible, degradable and environmentally friendly [135]. Aiming to reduce the cost, several raw biopolymers such as Okra [136], Gum Arabic, Exudate gum [137], Irvingia gabonensis [138], Kidney beans [139], etc., were investigated on their thickening effect.

#### 4.3.2. Filtration Properties

During the polymer flooding, filtration tests are designed to evaluate the pretreatment of polymers to prevent the injection well plugging and formation damage [140]. The plugging is due to the ineffective hydration of polymer caused by the poor agitation and slower addition rate, and the formation damage is caused by cellular debris or crosslinking of polymers catalyzed by the impurities in the polymer or in the makeup water. During the filtration tests, the filtration rate must be carefully controlled to mimic the injection rate as the deformed microgels may pass filtration tests but still cause the plugging problem later due to the lower shear rate in the real process [141]. Filtration tests also provide information about potential gelation issues triggered by the environmental factors and the necessity of controlling water quality. For example, Philips et al. applied the filtration tests to check the tolerance of high-pyruvate Xanthan to various ions and found the presence of ferric ion caused severe filterability deterioration [142].

#### 4.3.3. Polymer Flooding Compatibility

The performance of polymer flooding heavily depends on its compatibility on the oil viscosity, reservoir mineralogy, permeability, presence of clay, etc. According to the research conducted by Jewett and Schurz, biopolymer flooding commonly has promising results with an oil viscosity less than 126 mPa·s [143]. Moreover, the blending of Xanthan gum and polyacrylamide solution was used to deal with more viscous oil with a viscosity of up to 200 mPa·s. However, for viscous oil, the working concentration of polymer is extremely high, bringing expensive operation costs and high risk of failure [144]. The sandstone reservoirs are better candidates for polymer flooding than carbonate reservoirs, which lie in the fact that calcium ion of carbonate can increase the polymer adsorption on the formation, thereby reducing the effectiveness of flooding [145]. Based on the data of 46 polymer flooding projects, most of the 40 successful cases are sandstone reservoirs [146]. Du and Guan claimed that the polymer flooding was not recommended for the reservoir with permeability lower than 0.05 μm^2^ [147]. According to their observation, the injection of polymer solution under low permeability required an expensive pressure control and the high shear rate also damages the polymer viscosity due to both the shear-thinning phenomenon and polymer degradation. The presence of clay in the reservoir also deteriorates the performance of flooding as it may swell after contacting the injection fluid, resulting in a reduction in permeability.

Surfactant compatibility is another concern on designing polymer flooding, as polymer flooding is widely integrated with surfactant flooding to achieve better recovery rate. The combined effect of chemical and polymer flooding on oil recovery can be evaluated with the capillary number ($N_{ca}$), which is given by:

(8) $$N_{ca}=\frac{\mu_{w}\nu_{w}}{\sigma_{ow}}$$

where $\mu_{w}$ is the viscosity of water phase, $\nu_{w}\;$ is the volumetric fluid flux of water phase, and $\sigma_{ow}\;$ is the oil–water interfacial tension.

According to an empirical formula, a 1000-fold increase in the capillary number is expected to generate a reliable enhanced oil recovery process [148]. The polymer and surfactant bring a synergic effect as the former increase $\mu_{w}$ and the latter reduces the $\sigma_{ow}$. However, an improper mixing of polymer and surfactant could cause surfactant polymer incompatibility (SPI), which depends upon several factors such as the nature of head group of surfactant and the polar groups of polymer, as well as the polymer hydrophobicity [149]. SPI is detrimental to the efficiency of the oil recovery process as it causes the phase separation of surfactant and polymer molecules, increasing the surfactant loss due to colloidal aggregation and formation surface adsorption [148]. To avoid that, the polymer and surfactant screening needs to be conducted with various salinity to obtain a clear and stable aqueous solution.

#### 4.3.4. Polymer Flooding Stability

Various factors include salinity, temperature, shear rate, biodegradation and polymer retaining, which collectively affect the viscosity of polymer solution during the months, and even years, of water flooding. Compared with HPAM, biopolymers exhibit a higher tolerance of salinity, and additional chemicals can further improve their stability against salinity. For instance, Lachke found that the viscosity of low concentration Xanthan solution was stimulated by an additional 0.1% sodium or potassium chloride, as the ions can help polymer form a compact coiled structure with intermolecular association [150]. However, Xanthan solution is not stable at high salinity and loses 20–50% of viscosity in 20% salinity brine in days [151]. Temperature is another factor that affects the stability of biopolymer. Due to its strong intermolecular associations, Xanthan gum solution is believed to be stable at the temperature up to 80 °C. Alquraishi conducted the long-term stability study of Xanthan gum and found it can retain 50% of viscosity at 80 °C for more than five years [151]. The decrease in viscosity occurs when its double helix structure converts to a disordered coil, which happens extremely quickly at a temperature higher than 100 °C [152]. Interestingly, proper salt concentration can improve the thermostability of Xanthan. The absorption of salt ions on the polymer surface brings charges to the polymer, resulting in a repel force between molecules and inhibiting the structure collapse. Lund et al. reported Xanthan kept most of the viscosity at 90 °C with an additional 50 g/L NaCl [153]. When the polymer flooding is unavoidable at high temperatures and salinity, diutan and Scleroglucan are better candidates based on their supreme stability. For example, at 130 °C and 223 g/L salinity, injection of diutan gum solution realized a 19.34% oil recovery, compared with 14.15% of Xanthan gum flooding [154]. Quadri et al. also reported Scleroglucan can keep its entire viscosity at 135 °C and 220 g/L salinity over eight months [155]. The oxygen and bacterium fed on biopolymer are all unfavorable agents that affect the performance of flooding. The oxygen generates the hydroxyl radical (OH·) with the reaction of Fe^2+^, which can remove the acetate group in polymers and cause the degradation of polymers [101]. The growth of bacterium is also believed to cause both the degradation of polymers and unexpectable bio-plugging and formation damage. To address that, 25 to 100 ppm of formaldehyde is recommended to inhibit the growth of microbiology [156].

Besides degradation, the loss of biopolymer during the displacement is another aspect of polymer stability. The mechanisms of the loss include surface adsorption and mechanical entrapment. The surface adsorption is governed by the fluid pH and formation mineralogy. When the pH of fluid is below the isoelectric point of the mineral, a positive charge is expected on the surface of the rock, resulting in a high level of adsorption of anionic polymers. By controlling the fluid pH, the polymer adsorption can be less than 1 mg/g rock and the pH manipulation of flooding targeted to sandstone and carbonate reservoirs are different due to their varied isoelectric point (4.73 for kaolinite and 8.2 for limestone) [157]. Mechanical entrapment occurs when large molecules of the polymer are trapped in narrow flow channels, which can be reduced by utilizing biopolymer with smaller molecules.

### 4.4. Bio-Plugging

**Figure 13:**
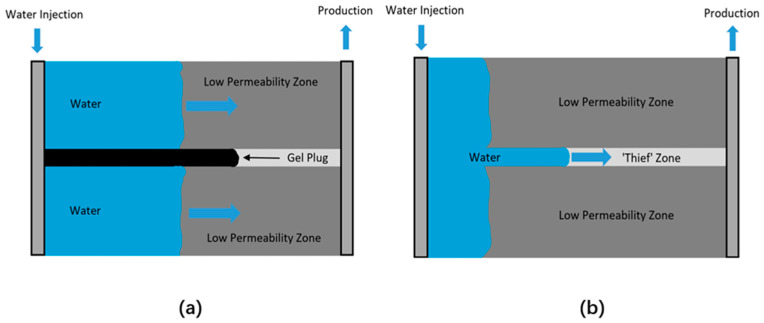
Water flooding of a reservoir with heterogeneous permeability with (**a**) or without (**b**) polymer plugging.

The initial application of biopolymer plugging was to deal with soil remediation. By the introduction of biopolymers as plugging agents, a large range of impervious barriers can be constructed to prevent the migration of environmental contaminations [158]. Biopolymers were also observed to react with soil particles to form cross-linking interpenetrating networks that can encapsulate the contaminants [159]. Inspiring from this concept, the plugging effect of polymer in petroleum production has been widely investigated since the 1990s [160,161,162]. The reservoir is comprised of several formation layers with various permeability, and the performance of water flooding is strongly affected by the permeability heterogeneity. When the layers are highly heterogenous in permeability, the injected water tends to flow only through the high-permeability “thief” zones, therefore failing to recover the oil that remains in the low-permeability zones [163]. To deal with it, the permeability of “thief” zones needs to be reduced to increase the water replacement in the low-permeability zones and the approach can be realized by using (bio)polymer gels, which is shown in Figure 13. Through accumulating in the microchannel of the formation, polymer plugs the pores of formation, reducing the permeability dramatically. The permeability reduction should be phase selective and water-based polymers reduce the water permeability more than the oil permeability. Based on the source of biopolymers, the plugging can be categorized as polymer-based and microbial-based.

#### 4.4.1. Gelled Biopolymer

Injecting linear polymer solution with crosslinker is a simple yet effective way to reduce the permeability of layers. By controlling the concentrations of polymer and crosslinker precisely, the gelation of biopolymer occurs. The gelled biopolymer can be deliberately triggered and accumulated in the target location with minimized friction during transportation. The Xanthan gum with Cr^3+^ system has been extensively applied in the plugging process due to its cost and efficiency [162]. The performance is highly affected by swelling and syneresis phenomenon. The former means the Xanthan can significantly increase its volume by absorbing water, and the latter describes the phenomenon that the swelled gel decreases its volume due to aging or continued crosslinking, resulting in a higher crosslink level and stiffness of the gel. As governed by chemical equilibrium [164], the severity of swelling and syneresis can be controlled by manipulating the concentration of crosslinker. For instance, Eggert et al. observed a 45% volume increase in Xanthan gel with 25 ppm of Cr^3+^, but a 68% volume decrease with 200 ppm of Cr^3+^. He also claimed that Xanthan gel should be controlled between 45% of swelling and 71% syneresis in order to achieve the best plugging effect and long-term stability [162]. The concentration of crosslinker was also believed to affect the selective penetration of biopolymer in the formation as weak gel commonly exhibited low retention and high mobility [165]. Besides the delayed gelation systems, pre-gels were also investigated on their plugging effect. Khachatoorian et al. studied the effect of various pre-gelled biopolymer on the permeability of sand park and reported decreases of 74.8%, 99.78%, 96.3%, 92.3% and 96.5% of permeability with 1 g/L of Xanthan gum, polyhydroxy butyrate, Guar gum, polyglutamic acid and Chitosan, respectively [166].

#### 4.4.2. Microbial Plugging

Due to the low cost, microbial plugging has been proposed as an economic method to manipulate the permeability. Through injecting nutrients, the bacterium can grow and block the pores of formation, resulting in a reduction in permeability. The reduction is selective to the thief zone as the growth rate of bacterium is higher there because of the sufficient nutrient supply. Both the applications of exogenous and indigenous bacterium species have been reported, and each species has its own optimal growth condition. Exhibiting a better plugging effect, exogenous species are preferred in the microbial plugging once the reservoir temperature fits its growth. In contrast, when the temperature is not suitable for exogenous species, injecting substrate to stimulate the indigenous targeted bacteria may also be helpful [167]. Microbial plugging has been successfully reported around the world. To reduce the unwanted water production during oil recovery, Reksidler et al. injected the microorganism as a slug, followed by 16-months of nutrient/electron acceptor injection. After the treatment, 4 out of 6 of the wells’ productions were improved and the reductions in water flow were between 10% and 60% [168]. In Daqing oilfield of North China, Le et al. conducted 518 wells of microbial huff-and-puff plugging and realized a cumulative oil increment of 1.2 × 10^5^ tons from 1998 to 2012 [169]. The plugging agent produced by bacterium includes bio-gel, biomass, biofilm, spore, or a combination, as shown in Table 8.

Before the field application, the performance of microbial plugging needs to be evaluated at the lab-scale to determine the best process strategy. The microbiology is cultured in the flask bottles at the reservoir temperature, and the growth of bacterium, as well as the production of the plugging agent, with various nutrition concentration is recorded and compared to determine the nutrition recipe. Then, the microbial plugging is conducted in a reservoir core or artificial micromodel to predict its effect of permeability reducing. Once the lab-scale results are promising, a single-well field test is executed, followed by a large-scale field application.

### 4.5. Wastewater Treatment

Oily water is considered as one of the serious public concerns during petroleum recovery due to its high volumes and toxic nature to the environment. Most countries have established strict regulations about the release of oily water and the discharge limit of oily wastewater is around 15–50 mg/L [179]. The oil is normally in the emulsified form, which makes the traditional phase separation method ineffective. Several advanced methods including liquid extraction, adsorption, hydrocyclones, air flotation, gravitational separators and filtration have been developed to deal with oily water [180]. Among these methods, the biopolymer-based approach attracts wide attention due to its reduced environmental impact. Chitosan and Guar gum are the main kinds of biopolymer dealing with oily water through adsorption and flocculation mechanism. Adsorption occurs when the oil passes through a microcrystalline or resinous of the polymer, remaining fixed at these sites due to the action of physical or chemical forces. As a partially deacetylated derivative, the amine groups of Chitosan have a high potential for adsorbing oil due to their high positive charge density. Ahmad and Sumathi achieved a 97–99% removal rate of oil from 2000 mg/L oily water in 5 min with 0.5 g/L of Chitosan and the performance was affected by the mixing rate, solution pH and sedimentation time [181]. Dai et al. applied a Guar gum coated stainless steel mesh for oil and water separation and got separate efficiency as high as 99.6% in a water flux of 2850 Lm^−2^h^−1^ [182]. To improve the adsorption capacity, the biopolymer can integrate with polar materials. For example, Wiltonet al. coated the biopolymer on the surface of polypropylene foam and exhibited a 94% removal of heavy hydrocarbon in a half-hour [183]. Flocculation occurs when the interaction of oil and polymer causes the aggregation of oil, and the charge neutralization is the main driving force [184]. Flocculation usually follows the adsorption process, making the removal easier and more effective. Paixão and Balaban designed a Guar gum-based approach to dealing with oily water: the oil was first adsorbed by the Guar gum, and then salt was added to trigger the flocculation. After the treatment, the oil concentration was reduced from 500 ppm to 11 ppm [185]. The synergy effect of biopolymer and surfactant on oil removal was also investigated by Calderón et al. In his work, sodium dodecyl sulfate (SDS) was added to surround the oil droplets, conferring negative charge, and bringing the binds between SDS and the polymer in the form of aggregates. The flocculation occurred in 3 h, resulting in very low turbidity values and a total hydrocarbon removal of 98.61% [186].

## 5. Conclusions

As the oil and gas will still be the dominant form of energy to support the development of the society, realizing an efficient and environmentally friendly petroleum recovery process is always a long-term goal of scientists and engineers. To address that, applying biopolymer as various fluid additives is helpful as it can improve the oil recovery, as well as reduce the cost of drilling, hydrofracking, and wastewater treatment. Compared with synthetic polymers, biopolymer exhibits more efficiency at a lower cost. However, the performance of biopolymer is strongly affected by the geological conditions and operation process. To deliver a successful biopolymer treatment, the recipe of biopolymer needs to be developed based on the special requirement of petroleum operation. As the functions of these biomacromolecules lie in their molecular conformations, characterizing and therefore modifying their structure is a promising approach to improving their thickening, crosslinking, and adsorption effects. Moreover, with the development of the modern fermentation process and biotechnology, the production cost of biopolymer is expected to decrease, increasing the economic feasibility of biopolymer in the petroleum recovery process.

## Figures and Tables

**Table 1:** Characteristics of biopolymers involved in petroleum recovery.

Biopolymer	Source	Monomers	Molecular Weight	Properties	Price (USD)	Modification	Ref.
Xanthan gum	Fermentation product of *Xanthomonas campestris*	D-mannose, D-glucose, Pyruvic acid, D-glucuronic acid	2 × 10^6^ to 2 × 10^7^ Da	Thickening Crosslinking	12/kg	Carbonate modified Formaldehyde modified Propylene oxide modified	[9,10,11,12,13,14,15,16,17,18]
Scleroglucan	Fermentation product of *Sclerotium rolfsii*	D-glucose	1.3 × 10^5^ to 6 × 10^6^ Da	Thickening	50/kg	Hydrophobic modified	[19,20,21,22,23,24]
Guar gum	Endosperm component of *Cyamopsis tetragonolobus*	D-mannose, D-galactose	10^6^ to 2 × 10^6^ Da	Thickening Crosslinking	2/kg	Hydroxypropyl modified Carboxymethyl modified Carboxymethyl hydroxypropyl modified	[25,26,27,28,29,30,31,32,33,34]
Cellulose	Lignocellulose of plants Fermentation product of *Acetobacter Xylinam*	D-glucose	2 × 10^6^ Da	Thickening Filtration Adsorption	4/kg	Hydroxyethyl modified Carboxymethyl modified Amphoteric modified	[35,36,37,38,39,40,41,42]
Chitin/Chitosan	Shells of crustaceans, exoskeletons of insects and cell walls of fungi	D-glucosamine, N-acetyl-D-glucosamine	2 × 10^3^ Da to 10^6^ Da	Adsorption	220/kg	Modification of *M*_W_	[43,44,45,46,47,48,49,50,51,52,53,54,55,56]

**Table 2:** The viscosity, *M*_W_ and polydispersity index of various carbonate-modified Xanthan gum solutions (0.5 wt%) (data from [16]).

Modification	Polymer: Carbonate Ratio	Viscosity (mPa·s) ^1^	*M*_W_ (Da)	Polydispersity Index
Control	NA	680	3.98 × 10^6^	1.25
Ethylene Carbonate	1:0.132	1640	5.89 × 10^6^	1.06
Propylene Carbonate	1:0.132	1040	7.0 × 10^6^	1.32
Butylene Carbonate	1:0.122	1040	4.44 × 10^6^	1.26
Diethyl Carbonate	1:0.122	1000	5.67 × 10^6^	1.24
Glycerine Carbonate	1:0.130	2200	6.93 × 10^6^	1.50

^1^ At 50 s^−1^ shear rate and room temperature.

**Table 3:** The rheology properties of drilling fluids with biopolymer.

Recipe	Model	Parameters	Ref.
Xanthan gum, starch and bactericide and clay were 5.75, 11.5, and 1.72 g/L, respectively, then 10 wt% clay was added.	Herschel–Bulkley	$\tau_{0}$: 3.78 (Pa) K: 3.22 (Pa·s ^n^) n: 0.31	[83]
Scleroglucan, starch and bactericide were 5.75, 11.5, and 1.72 g/L, respectively, then 10 w/w % clay was added.	Herschel–Bulkley	$\tau_{0}$: 3.36 (Pa) K: 0.79 (Pa·s ^n^) n: 0.72	[83]
Xanthan gum, starch, NaCl, paraformaldehyde and clay were 0.5, 1.5, 0.75, 0.125 and 2.5 wt%, respectively.	Herschel–Bulkley	$\tau_{0}$: 3.88 (Pa) K: 0.46 (Pa·s ^n^) n: 0.64	[84]
Cellulose nanoparticles, bentonite and polyanionic cellulose were 3.05, 10.15, and 0.87 g/L, respectively.	Herschel–Bulkley	$\tau_{0}$: 0.41 (Pa) K: 0.44 (Pa·s ^n^) n: 0.53	[85]
Water contains 5 g/L of Xanthan gum.	Casson	$\tau_{0}$: 6.32 (Pa) $\mu_{p}$: 0.58 (10^−3^ mPa·s)	[86]
Cellulose nanoparticles, bentonite and polyanionic cellulose were 0.5, 4.5, and 0.05 g/L, respectively.	Casson	$\tau_{0}$: 3.43 (Pa) $\mu_{p}$: 0.13 (10^−3^ mPa·s)	[87]
Water contains 1 g/L Lepidium perfoliatum seed gum.	Casson	$\tau_{0}$: 10.31 (Pa) $\mu_{p}$: 0.23 (10^−3^ mPa·s)	[88]

**Table 4:** The viscosity of biopolymer under various condition.

Polymer Type	Concentration (wt%)	Temperature (°C)	Shear Rate (s^−1^)	Viscosity (mPa·s)	Ref.
Guar gum	0.24	25	511	10	[108]
	0.54	25	511	42	
	0.95	25	511	103	
	0.48	25	10	250	[109]
	0.48	25	100	88	
	0.48	25	1000	24	
	1	25	15	225	[110]
	1	40	15	160	
	1	60	15	120	
	1	80	15	80	
CMHPG	0.48	25	170	58	[111]
	0.48	25	511	35	

**Table 5:** The crosslinkers of Guar gum solution.

Type	Form	Bond	pH	Temperature (°C)
Borate	Borax; Boric acid	Hydrogen; Ionic	8–11	38–107
Ti^4+^	Titanium acetylacetonate Titanium mono-triethanolamine chelate	Covalent bond	3–11	38–163
Zr^4+^	Zirconium ammonium lactate Zirconium tetra-acetate	Covalent bond	3–11	38–177
Al^3+^	Aluminum phosphate	Covalent bond	3–5	38–94

**Table 6:** The breakers of hydraulic fluid.

Category	Form	Disadvantages	Advantages
Enzymes	Hemicellulose	Unstable when T > 135 °C or pH > 10.5	Environmentally benign Reaction specific Effective Leave less residue
Oxidizers	Ammonium, potassium sodium peroxydisulfate	Slow when T < 52 °C Harm to equipment	Tolerance of high temperature

**Table 7:** Rheology parameters of biopolymers and HPAM.

Polymer Type	Concentration (%)	n	*K* (m Pa·s ^n^)	Ref.
HPAM	1	0.28	1080	[129]
	2	0.26	2050	
	5	0.25	5770	
Xanthan gum	0.5	0.58	1190	[130]
	1	0.65	3163	
	2	0.71	6526	
Scleroglucan	0.5	0.49	55	[19]
	1	0.31	272	
	2	0.20	1073	
CMC	1	0.95	50	[131]
	2	0.85	450	
	4	0.61	830	

**Table 8:** Examples of permeability manipulation with microbial plugging.

Microbial Species	Plugging Agents	Results	Ref.
*Leuconostoc mesenteroides*	Bacterial dextran	Permeability decreased from 4.08 μm^2^ to 0.17 μm^2^ and a 36.7% improvement of the oil recovery in lab scale	[170]
*Bacillus licheniformis* BNP29	Biomass, Extracellular polymer	A 9.3–22.1% additional recovery of the residual oil after water flooding	[171]
*Enterobacter* sp. CJF002	Insoluble biopolymer	A 260% increase in oil production in field test	[172]
*B. licheniformis* 421	Spore	Additional 1.0–2.3% and 6.9–8.8% oil recovery in homogenous and heterogeneous reservoir chalk cores, respectively	[173]
*Pseudomonas* sp.	Exopolysaccharides, biofilm	A more than 99% decrease in core permeability	[174]
*Bacillus licheniformis* TT33	Biofilm, Biopolymer	A 20–30% additional oil recovery in a sand pack column	[175]
B3 bacterium isolated from reservoirs of Carmopólis field	Biopolymer	A 20% additional oil recovery in the laboratory test	[176]
*Shewanella oneidensis* MR-1	Biofilm	A 7.1% additional oil recovery after water flooding in microfluidic channels	[177]
*Acinetobacter* RAG-1	Biofilm	A 18% additional oil recovery after a 41% oil recovery from water flooding in micromodel	[178]
